# Supplementary material for: Monitoring and Genotyping of Wild Grapevine (Vitis vinifera L. subsp. sylvestris) in Slovenia
Source: Plants (Basel). 2024 Apr 29;13(9):1234. doi: 10.3390/plants13091234 (PMC11085864; doi:10.3390/plants13091234)
Supplement: Supplementary file 1 [file plants-13-01234-s001.zip › Figure_S2.pdf]

Figure S2: Best K for *sylvestris* populations

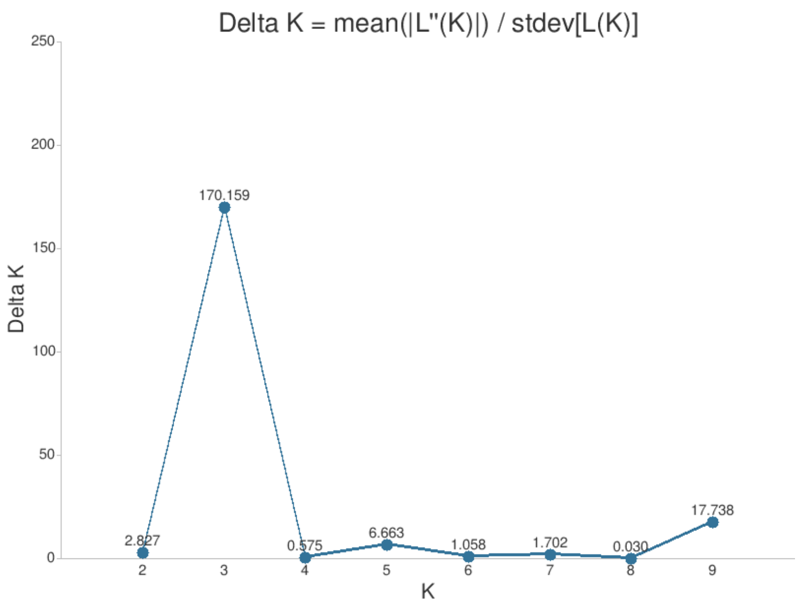

(a)

Results of STRUCTURE analyses. Calculating Best K by Evanno. Three different genetic groups (populations) were suggested (ΔK = 3). The test was run from K = 1 to 10 using STRUCTURE software.
